# A precocious adult visual center in the larva defines the unique optic lobe of the split-eyed whirligig beetle *Dineutus sublineatus*

**DOI:** 10.1186/1742-9994-10-7

**Published:** 2013-02-19

**Authors:** Chan Lin, Nicholas J Strausfeld

**Affiliations:** 1 Graduate Interdisciplinary Program in Entomology & Insect Science, University of Arizona, Tucson, AZ, 85721, USA; 2 Center for Insect Science, University of Arizona, Tucson, AZ, 85721, USA; 3 Department of Neuroscience, University of Arizona, Tucson, AZ, 85721, USA

**Keywords:** Gyrinidae, Optic lobe, Lobula plate, Motion detection, Stemmata, *Cicindela*

## Abstract

**Introduction:**

Whirligig beetles (Coleoptera: Gyrinidae) are aquatic insects living on the water surface. They are equipped with four compound eyes, an upper pair viewing above the water surface and a lower submerged pair viewing beneath the water surface, but little is known about how their visual brain centers (optic lobes) are organized to serve such unusual eyes. We show here, for the first time, the peculiar optic lobe organization of the larval and adult whirligig beetle *Dineutus sublineatus*.

**Results:**

The divided compound eyes of adult whirligig beetles supply optic lobes that are split into two halves, an upper half and lower half, comprising an upper and lower lamina, an upper and lower medulla and a bilobed partially split lobula. However, the lobula plate, a neuropil that in flies is known to be involved in mediating stabilized flight, exists only in conjunction with the lower lobe of the lobula. We show that, as in another group of predatory beetle larvae, in the whirligig beetle the aquatic larva precociously develops a lobula plate equipped with wide-field neurons. It is supplied by three larval laminas serving the three dorsal larval stemmata, which are adjacent to the developing upper compound eye.

**Conclusions:**

In adult whirligig beetles, dual optic neuropils serve the upper aerial eyes and the lower subaquatic eyes. The exception is the lobula plate. A lobula plate develops precociously in the larva where it is supplied by inputs from three larval stemmata that have a frontal-upper field of view, in which contrasting objects such as prey items trigger a body lunge and mandibular grasp. This precocious lobula plate is lost during pupal metamorphosis, whereas another lobula plate develops normally during metamorphosis and in the adult is associated with the lower eye. The different roles of the upper and lower lobula plates in supporting, respectively, larval predation and adult optokinetic balance are discussed. Precocious development of the upper lobula plate represents convergent evolution of an ambush hunting lifestyle, as exemplified by the terrestrial larvae of tiger beetles (Cicindelinae), in which activation of neurons in their precocious lobula plates, each serving two large larval stemmata, releases reflex body extension and mandibular grasp.

## Introduction

In insects, the optic lobes consist of three nested neuropils beneath the photoreceptive retina: the lamina, the medulla and the lobula complex. In coleopterans, as in several other insect orders, the lobula complex is divided into two discrete neuropils, a lobula and a lobula plate. The lamina, medulla and lobula are columnar neuropils sequentially linked by optic chiasmata, whereas the planar lobula plate receives uncrossed axons from the medulla and lobula and is characterized by wide-field tangential neurons [1]. Studies on dipterous insects have shown that these neurons, often having very large axon diameters, are tuned to visual flow-field stimuli by virtue of integration of signals carried by small-field directional motion-sensitive neurons originating retinotopically in the lobula and medulla [2]. Along with relays from the lamina and medulla, which carry achromatic information to these retinotopic neurons [3], this system comprises the most direct and thus fastest pathway in the optic lobes and is comparable to the color-insensitive magnocellular system of the vertebrate retina [4].

Although lobula plate tangential neurons are usually considered part of an adult circuit that contributes to the stabilization of flight, observations of the predatory larvae of tiger beetles (Cicindelinae) suggest that this neuropil can develop in the larva. Relays from two pairs of large single-lens eyes (stemmata), via an underlying larval lamina neuropil, supply a reniform neuropil (called the tectum [1]) equipped with wide-field tangential neurons that are tuned to movement of prey-sized objects across the opening of the larva’s burrow [5-8]. Responses by these neurons result in reflex-like extensions of the larva’s body and a mandibular grasp culminating in prey capture [9,10]. At metamorphosis, the cicindelid stemmata degenerate, as do their photoreceptor axons. This results in a cascade of subsequent degeneration: first the larval lamina and then the underlying tectum containing wide-field neurons. On eclosion, the adult tiger beetle is equipped with compound eyes, each supplying a lamina, medulla, and lobula [1]. But the lobula plate, which is usually present in other terrestrial coleopterans, is absent. The larval tectum has thus been interpreted as resulting from the precocious development of the adult lobula plate, which is employed in the larva for visually mediated predation [1].

Is precocious use of an adult visual neuropil unique to tiger beetles? Might other predatory larvae use similar strategies? Here we provide evidence that at least one other coleopteran taxon has evolved this developmental strategy: the whirligig beetle *Dineutus sublineatus* (Coleoptera: Gyrinidae), the larvae of which are subaquatic ambush predators. Adult whirligig beetles are aquatic insects living on the water surface. They are equipped with four compound eyes, an upper (aerial) pair viewing above the water surface and a lower (aquatic) pair viewing beneath the water surface. The upper and lower eyes supply a separate lamina and medulla, relays from which supply a bilobed lobula. A previous description demonstrated that a unique attribute of the upper eye medulla is its relays supplying exclusively the visual calyces of the paired mushroom bodies [11]. Here we describe a further peculiarity of this optic lobe, which is the presence of a lobula plate exclusively serving the lower eye. A lobula plate serving the upper eye is absent. Observations of the habits of the whirligig larva and its visual system resolve the likely cause of this deficit.

## Results

### Split optic lobes of the adult whirligig beetle *D. sublineatus*

**Figure 1:**
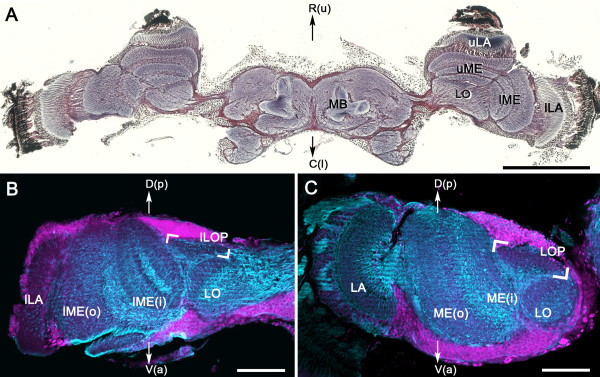
**Brain and optic lobe organization in the adult whirligig beetle *Dineutus sublineatus *and the sunburst diving beetle *Thermonectus marmoratus. *A**: The split optic lobes of the whirligig beetle, including upper and lower laminas (uLA and lLA), medullas (uME and lME) and a bilobed lobula that is fused at this frontal level of section (LO). **B**, **C**: Comparison between the whirligig lower optic lobe (**B**) and the optic lobe of the sunburst diving beetle (**C**). Each is equipped with a comparable lamina (LA), medulla (ME, outer and inner layers), lobula (LO) and a tectum-shaped lobula plate (LOP, in brackets). Blue: α-tubulin immunoreactivity; magenta: SYTO 13 nuclear staining. **A**: frontal section with the rostral brain margin to the top (rostral R according to the neuraxis = upper (u) according to body axis; caudal C according to the neuraxis = lower (l) according to body axis); **B**, **C**: horizontal section with the posterior brain margin to the top (D, neuraxis dorsal; p, body axis posterior; V, neuraxis ventral; a, body axis anterior). MB, mushroom body. Scale bars = 1mm in **A**; 100 μm in **B**, **C**.

**Figure 2:**
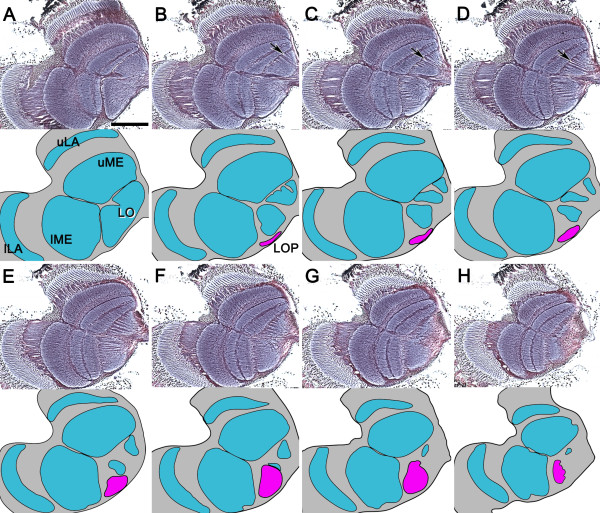
**The lobula plate is present only in the lower optic lobe in the adult whirligig beetle. A-H**: Eight consecutive reduced silver-stained frontal brain sections taken from the anterior to posterior optic lobe, and the schematic drawings pertaining to each level, demonstrate the position of the lobula plate (LOP, magenta), which first appears just beneath the lower part of the lobula (**B-D**) and is then revealed to be restricted to the lower half of the optic lobe (**E-H**). The upper lobe of the lobula is fragmented by small parallel bundles of axons that project directly from the upper medulla towards the medial protocerebrum (**B-D**, arrows). uLA and lLA, upper and lower lamina; uME and lME, upper and lower medulla; LO, lobula. All panels are of the same magnification (bar in **A** = 200 μm).

The compound eyes of whirligig beetles are divided into two parts, one half viewing the aerial scene, the other viewing beneath the waterline. Reduced silver-stained frontal sections show the characteristic columnar organization of retinotopically arranged neurons in the underlying optic lobes. The laminas and medullas are composed of as many columns as there are ommatidia, whereas in the lobula the periodicity of columnar neurons is coarsened, as it is in many other insect species. These basic arrangements in *D. sublineatus* are independent of its split optic lobes (Figure 1A). The lobula appears fused in its most anterior parts (Figure 1A), but is separated into an upper and lower lobe more posteriorly (Figure 2). Each lobe receives its inputs from the corresponding upper and lower medullas and these receive their peripheral inputs from the corresponding upper and lower laminas.

As is typical for coleopterous insects, the lobula complex is divided into a lobula and a planar stratified neuropil, the lobula plate. As in other insect orders that are similarly endowed, the lobula plate lies posterior to the lobula (dorsal to the lobula according to the neuraxis; Figure 1B,C). However, in whirligig beetles, lobula plate neuropil is present only posterior to the lower lobe of the lobula, as shown in an immunostained horizontal section of the lower eye (ILOP in Figure 1B). There is no corresponding lobula plate associated with the upper lobe of the lobula (Figure 2). Figures 1B and C compare the organization between optic lobe neuropils serving the whirligig’s lower eye (Figure 1B) and an optic lobe serving the whole eye of the adult sunburst diving beetle *Thermonectus marmoratus* (Figure 1C). Both optic lobes possess comparable laminas, medullas (divided into outer and inner layers), lobulas, and lobula plates. In both species, the medulla receives crossed inputs from the lamina, and the lobula receives crossed inputs from the medulla. The lobula plate receives uncrossed inputs separately from the medulla and lobula. Thus, optic lobe organization of the eyes (the lower compound eye of *D. sublineatus* and the entire compound eye of *T. marmoratus*) conforms to the terrestrial coleopteran ground pattern.

### Lobula plate neuropil in the lower half of the *D. sublineatus* optic lobe

Absence of lobula plate neuropil posterior to the upper lobe of the lobula can be best demonstrated in a series of consecutive frontal brain sections. As illustrated in Figure 2, eight of the sections show the split upper and lower laminas (uLA and lLA) and medullas (uME and lME). The upper and lower lobes of the lobula are fused anteriorly (Figure 2A), but are separated more posteriorly (Figure 2B-F). The first evidence of a discrete lobula plate neuropil is an aerofoil-shaped structure immediately beneath the lower part of the undivided lobula (Figure 2B, LOP shown magenta). This structure enlarges and expands upwards more posteriorly behind the lower lobe of the lobula. At this level, the lenticular-shaped lobula plate receives uncrossed parallel inputs from the lower medulla (Figure 2E-H). Lobula plate neuropil never extends across the equator demarcating the upper and lower halves of the optic lobe. This means that the lobula plate can only serve the lower aquatic eye. Posteriorly, the upper lobe of the lobula appears split into smaller volumes imposed by parallel bundles of axons (Figure 2C,D, arrows). These axons originate from the upper medulla and project inwards to the medial protocerebrum and then to the mushroom body calyces [11]. These observations suggest that in the adult whirligig beetle there are distinct functional differences between the upper aerial eyes and the lower aquatic eyes.

### The whirligig larval visual system

**Figure 3:**
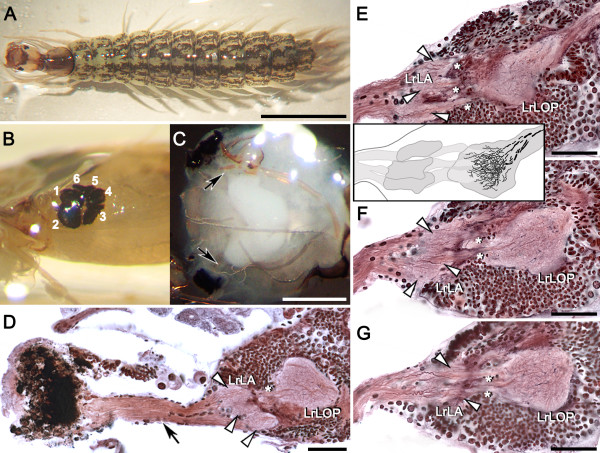
**The visual system of 3**^**rd **^**instar whirligig beetle larva. A**: A 3^rd^ instar larva. **B**: Group of six stemmata located laterally on the head. **C**: Top-down view of the brain after removing the head capsule. Larval stemmata are connected to the brain by a fused stemmatal nerve (bundles of retinular axons, arrows). **D**: A horizontal silver-stained section showing the stemmatal nerve (black arrow), three major larval laminas (LrLA, white arrowheads) and precocious larval lobula plate (LrLOP). The lobula plate is supplied by uncrossed axons from the lamina (asterisk). **E-G**: Three consecutive horizontal brain sections of another preparation showing the three larval laminas (LrLA, white arrowheads) and the larval lobula plate (LrLOP), which receives uncrossed inputs from the laminas (asterisks). **Inset F**: Reconstruction from serial Bodian-stained sections of the larval laminas and lobula plate showing dendrites and centrally projecting axons. Scale bars = 5 mm in **A**; 500 μm in **C**; 50 μm in **D-G**.

Whirligig larvae (Figure 3A) are subaquatic ambush predators preying on small arthropods using a lunge-and-snap action of their pincer-like mandibles. In the laboratory’s aquaria, larvae were observed hiding in gravel on the bottom and attacking prey above them (freshly thawed bloodworms *Chironomus* sp.). This behavior is interpreted as being triggered by visual stimuli: the bloodworms were immobile, providing no mechanical stimuli, and the beetle larvae are thought to be anosmic. Like the larvae of many holometabolous insects, whirligig larvae are equipped, on each side of the head, with six single-lens stemmata, three of which view an upper frontal visual field. The six stemmata on one side of the head can be best seen from a lateral view in Figure 3B. Photoreceptor axons under each stemma bundle together and project inwards to the larval brain (Figure 3C,D, arrows). The larval brain has distinct optic lobes that comprise three prominent neuropils, here referred to as larval laminas (Figure 3D, arrowheads), and a deeper orbicular neuropil. Consecutive sections clearly show three spatula-shaped laminas supplied by three distinct bundles of photoreceptor axons (Figure 3E-G, arrowheads). The three laminas send uncrossed axons to the deeper orbicular neuropil (Figure 3E-G, asterisks). This uncrossed arrangement is characteristic of a lobula plate that, in the adult, would normally receive uncrossed inputs from the medulla and lobula [1]. In the orbicular neuropil of the larva, extensive wide-field processes are interspersed amongst the incoming parallel inputs and send bundled axons centrally to the brain (Figure 3E-G, and inset to F). Wide-field tangential neurons are characteristic of adult lobula plates, where they respond to visual motion [2,3]. Together, these observations indicate that visual information in whirligig larvae is processed by a well-developed optic lobe that consists of larval laminas supplying a lobula plate-like center. This feature likewise characterizes tiger beetle larvae, which are also ambush hunters [5-10].

### The larval lobula plate is supplied by relays from the three dorsal stemmata

**Figure 4:**
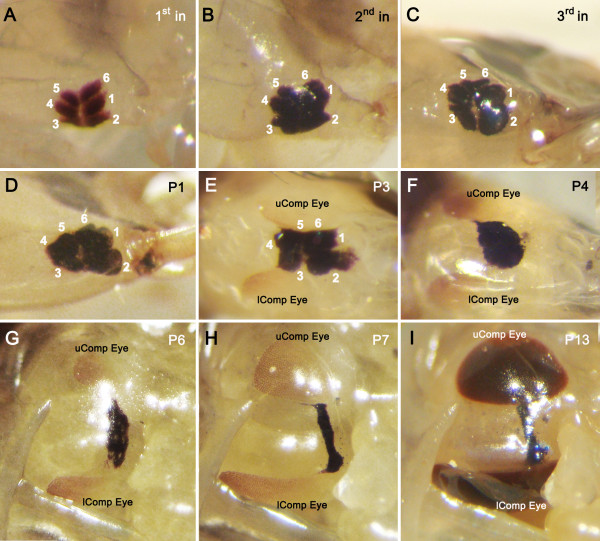
**External characteristics of larval stemmata and developing adult compound eyes during the immature stages of the whirligig beetle. A-C**: The morphology and disposition of six stemmata of one side of the head in each of the three larval instars (1^st^ -3^rd^ in). Stemmata 1 and 2 become comparatively larger and stemmata 5 and 6 become comparatively smaller in the 3^rd^ instar. **D-I**: During pupation, the larval stemmata gradually degenerate and the adult upper and lower compound eyes (uComp and lComp Eye) are gradually formed on the dorsoposterior and ventroposterior side of the larval stemmata. P1-13, day 1–13 of pupation at room temperature.

**Figure 5:**
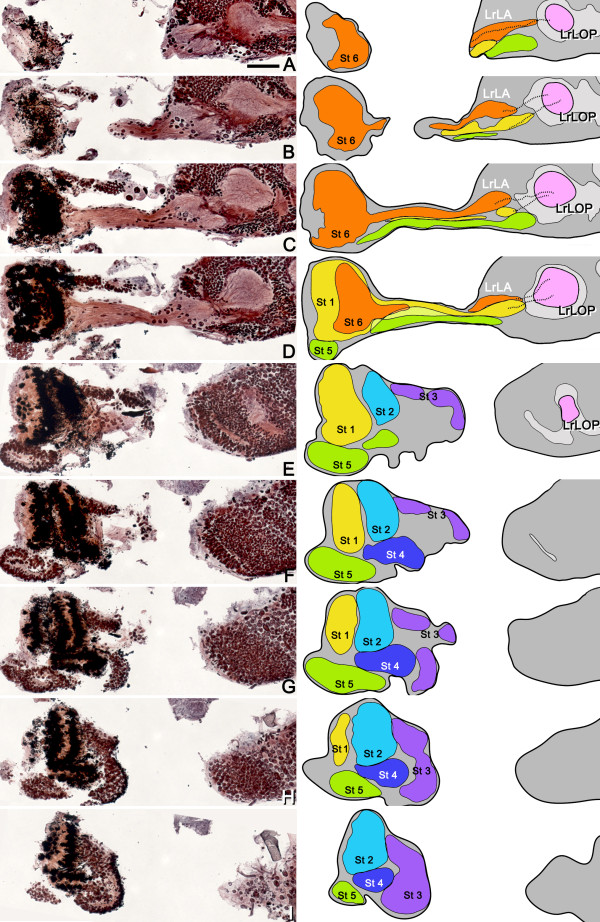
**The precocious lobula plate is supplied by the dorsal three stemmata, but not the ventral three stemmata, of the larval whirligig beetle. A-I**: Nine consecutive reduced silver-stained horizontal sections of the 3^rd^ instar larval visual system from dorsal to ventral, and their corresponding schematic drawings, show the three larval laminas (LrLA) and the precocious lobula plate (LrLOP, pink) supplied from the dorsal three stemmata (St6, orange; St1, yellow; St5, green), but not the ventral three stemmata (St2, teal; St4, blue; St3, purple). All panels are of the same magnification (bar in **A** = 50 μm).

In holometabolous insects, throughout pupal metamorphosis and continuing into the adult stage, photoreceptor axons projecting into the brain from larval stemmata serve as guideposts for the ingrowth of developing photoreceptor axons of what will become the adult compound eyes [12,13]. Interneurons belonging to larval optic neuropils, if present, have been suggested to provide templates for the development of corresponding neuropils that will become fully operational in the adult [12]. Why, then, in the adult whirligig beetle is the upper eye’s lobula plate absent? To determine if this absence reflects the relationship of the developing upper eye with larval stemmata supplying relays to the lobula plate-like orbicular neuropil described in the previous section, we examined the external characteristics of the stemmata throughout their larval and pupal stages, subsequently focusing on the neuroanatomy of the 3rd instar visual system. Figure 4 shows the disposition of the six stemmata of one side of the head at different immature stages. First instar larvae possess six approximately equal-sized stemmata on each side of the head, consisting of a front row (Figure 4A, stemmata 1, 2 and 6) and a back row (Figure 4A, stemmata 3–5). In the 2^nd^ and 3^rd^ instar larvae, stemmata 5 and 6 become progressively smaller whereas stemmata 1 and 2 become larger (Figure 4B,C). During pupation, the larval stemmata slowly degenerate (Figure 4D-I) and the adult upper and lower compound eyes develop above and beneath the dorsoposterior and ventroposterior margins of the diminishing larval stemmata (Figure 4E-I, uComp Eye and lComp Eye). Thus, stemmata 1, 5 and 6 are physically closer to the developing adult upper compound eye (Figure 4C-E), whereas stemmata 2, 3 and 4 are physically closer to the adult lower compound eye (Figure 4C-E). Figure 5 shows a series of nine consecutive horizontal sections of the 3^rd^ instar whirligig larval visual system demonstrating the relationship of the orbicular larval neuropil with stemmata 1, 5 and 6. Serial sections, and the corresponding schematic drawings, show that the orbicular neuropil in the larval optic lobe (Figure 5A-E, LrLOP) is supplied by bundles of uncrossed axons from the three spatulate larval laminas serving the three upwardly viewing stemmata 1, 5 and 6 (Figure 5A-D, LrLA). The ventral downwardly viewing stemmata 2, 3 and 4 supply axons that extend inwards to optic lobe Anlage that will provide the adult lamina, medulla and lobula complex, but they do not appear to supply any larval neuropils (Figure 5E-I).

Stemmatal positions are therefore of crucial importance for interpreting adult optic lobe organization. Only the laminas supplied from the dorsal three stemmata, 1, 5 and 6, relate to the underlying lobula plate-like neuropil. Because the three dorsal-most stemmata are physically closest to the developing upper compound eye, this arrangement supports the hypothesis that a lobula plate that would otherwise subtend the upper compound eye in the adult beetle indeed develops precociously in the larva where it serves to process information relayed from those upwardly viewing stemmata. The larval lobula plate neuropil does not survive metamorphosis, nor does it provide a “template” for the development of an adult lobula plate serving the upper eye; that neuropil is lacking in the imago.

## Discussion

### The optic neuropils of larval holometabolous insects

The larval visual system of holometabolous insects consists of six single-lens stemmata on each side of the head. These have evolved to various degrees of complexity [12]. However, compared with what has been documented for the optic lobes in adult insects, much less information is available on the organization of optic neuropils and their circuitry serving larval stemmata. There are exceptions, however. In the fruit fly *Drosophila melanogaster*, larval stemmata are reduced to just 12 photoreceptors that provide inputs for simple phototactic behaviors, classical conditioning [14], and the control of circadian rhythms [15]. Photoreceptor axons project into a small stalk-like larval optic neuropil where they directly synapse onto lateral clock neurons involved in the entrainment of the circadian molecular clock and onto the optic lobe pioneer neurons that subsequently give rise to the adult optic lobes [16,17]. Neither columnar optic neuropils nor true visual interneurons have been found in the larva, however. This contrasts with chaoborid and culicid Diptera, whose aquatic larvae are highly mobile and are visually efficient in mediating predator avoidance behavior [12]. In *Chaoborus crystallinus*, peramorphic (adult-like) compound eyes have been reported in larval and pupal stages [18]. Developing ommatidia of what will become the adult compound eye are connected to a columnar lamina connected by a chiasma to a columnar medulla. There are also two successive larval neuropils that are associated with persisting larval stemmata [19].

The most sophisticated stemmatal larval eyes have been reported in the larvae of adephagan Coleoptera, including the predatory sunburst diving beetle *T. marmoratus* and tiger beetles, such as *Cicindela chinensis*. In the predaceous larval *T. marmoratus*, two large tubular stemmata and four smaller spherical stemmata on each side of the head are equipped with distinctive arrangements of 28 asymmetric retinas [20]. Stemmatal photoreceptors from these retinas supply a larval optic lobe consisting of two exceptionally large neuropils and four smaller ones, each receiving receptor inputs from one of the stemmata [20,21]. The two largest of these larval laminas have columnar arrangements of receptor endings that intersect layers [21]. The layers likely comprise processes of stratified interneurons that are likely to integrate information from the several retinas thereby mediating target detection by a larva that employs characteristic vertical scanning movements to locate and seize its prey [22]. At metamorphosis, larval laminas degenerate and are substituted by the nested optic lobes of the adult beetle [21].

Of particular interest are larval tiger beetles, which are ambush predators that make their burrows in sandy ground. Depending on the size and vertical distance relative to the burrow’s opening of an object passing over it, the larva exhibits either a predatory jump-and-snap, or it withdraws and hides deep within the burrow [9,10,23,24]. Two enlarged stemmata located dorsally on each side of the head mediate this behavior [7,10,23]. Each of the two stemmata has 4,000 – 5,000 retinular cells, the bundled axons of which project to an enlarged composite optic lobe composed of two nested neuropils, two adjacent regions lying distal to a large reniform neuropil beneath [1,8,25]. Toh & Mizutani [25] characterized four types of monopolar interneurons relaying from photoreceptor axon terminals in the first neuropils, which they called the larval “laminas”, to the second neuropil that they termed the larval “medulla”. However, because the monopolar neurons project uncrossed axons from the larval laminas to the second neuropil [8,25], and because of its complement of large wide-field tangential neurons tuned to motion [5-7], it was proposed that this second neuropil is likely to be a precocious lobula plate [1]. Examination of the adult optic lobes demonstrated the absence of the lobula plate in the imago [1]. During pupal metamorphosis, the larval stemmata and the underlying lamina’s neural circuitry degenerate, as does the deeper reniform neuropil supplied by the larval laminas.

### Ambush hunting lifestyle and precocious lobula plates in tiger beetle and whirligig beetle larvae

**Figure 6:**
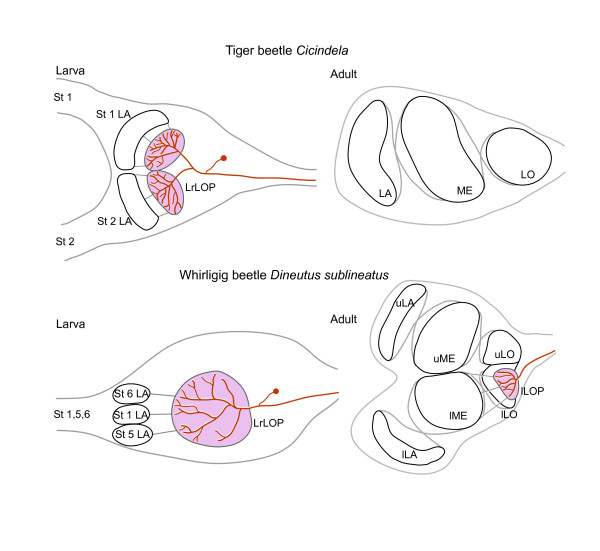
**Schematics comparing the larval and adult optic lobes of the tiger beetle *Cicindela *(after [**[1,][5]**]) and the whirligig beetle *D. sublineatus. *Upper and lower left**: Both larvae are ambush predators and are equipped with precociously developed larval lobula plates (LrLOP, pink) that contain wide-field neurons supplied by uncrossed axons from prominent stemmatal laminas (St LA). These receive their inputs from the dorsal-most stemmata in tiger beetle larva (St 1 and 2) and whirligig larva (St 1, 5 and 6). **Upper and lower right**: During pupal metamorphosis, larval stemmata and their underlying optic neuropils degenerate. On eclosion, the adult tiger beetle possesses only a lamina (LA), medulla (ME), and lobula (LO), but lacks the lobula plate. In the adult whirligig beetle, the lower lobula plate (lLOP, pink) serving the lower aquatic eye develops normally, but the upper lobula plate is absent.

Figure 6 illustrates the comparable optic lobe organization between the larval and adult stages of tiger beetles and whirligig beetles. In both taxa, uncrossed axons from the larval laminas terminate amongst the processes of wide field neurons in what we interpret as precocious lobula plates. In tiger beetle larvae, the lobula plate is partially split into two parts, each receiving inputs from one of the laminas. Electrophysiological studies of *C. chinensis* have demonstrated that motion detecting wide-field tangential neurons extend their dendrites either within one or both parts of the lobula plate [5,7]. In contrast, the precocious lobula plate in *D. sublineatus* is an undivided neuropil that receives uncrossed inputs from the three laminas subtending the three upward-looking stemmata (Figure 3D-G, Figure 6 lower left). Although both of these coleopterans belong to Adephaga, the number and dispositions of their stemmata and larval laminas, and the differences in their lobula plate morphologies, suggest that the presence in both taxa of a precocious lobula plate does not derive from a common ancestor that possessed this peramorphic character but is the result of convergent evolution of homoplastic arrangements that support two distinctive visually guided ambush behaviors. In contrast, observations of the visual system of the larval sunburst diving beetle *T. marmoratus*, which also belongs to Adephaga and which exhibits visually guided scanning behaviors for detecting prey [22], lack- any evidence of a lobula plate-like neuropil central to its six stemmatal laminas [21].

In both whirligig and tiger beetles, the precocious larval lobula plates degenerate during pupal metamorphosis. As a consequence, the adult tiger beetle has no lobula plate, whereas the adult whirligig beetle lacks the lobula plate neuropil subtending the upper compound eye only (Figure 6).

### Functional implications of the lower lobula plate in the adult whirligig beetles

In Lepidoptera, Coleoptera and Diptera, the adult lobula plate is hallmarked by wide-field tangential neurons [1]. In Lepidoptera and Diptera, such neurons respond predominantly to visual stimuli moving across the compound retina [2,26,27]. The lobula plate tangential neurons supply inputs to descending neurons that project to the thoracic ganglia where they supply motor circuits participating in the activation and control of flight and head movement [28,29]. The essential role of the lobula plate is therefore to integrate visual flow field information and contribute to stabilized flight [30]. Like their aerial counterparts, lobula plates in adult aquatic coleopterans, such as the sunburst diving beetle *T. marmoratus*[21], are likely to possess motion detection circuits that serve comparable functions for motion stabilization in water. Indeed, body stabilization is as crucial for a swimming coleopteran as it is for an insect flying in the air. Because the lobula plate of the adult whirligig beetle *D. sublineatus* serves only the lower (aquatic) eyes, this suggests that those eyes alone contribute to locomotory balance under water. Their contribution to aerial flight is likely to be negligible: flight by a whirligig beetle is a rarity.

## Conclusions

Here we have shown that in the elaborate visual brain of the four-eyed whirligig beetle *D. sublineatus*, each optic neuropil is divided into an upper and lower lamina, and an upper and lower medulla, together supplying a bilobed lobula. The exception is the lobula plate. The lobula plates serving the upper and lower eyes develop at different developmental stages. The upper lobula plate develops precociously in the larva and supports ambush hunting. The lower lobula plate develops during metamorphosis and is present in the imago where it likely supports subaquatic locomotory balance. Precocious development of the lobula plates in tiger beetles and whirligig beetles suggests convergent evolution of larval visual systems for an ambush hunting lifestyle.

## Materials and methods

### Insects

About 40 adult whirligig beetles (*Dineutus sublineatus*) and 20 sunburst diving beetles (*Thermonectus marmoratus*) were collected at Sycamore Canyon, Nogales, Arizona, and maintained in separate tanks (90x30x35 cm) in the laboratory at 24°C. They were fed daily with frozen fruit flies sprinkled onto the water surface and freshly thawed bloodworms (*Chironomous* sp.; Hikari Bio-Pure, Hayward, Calif.). Styrofoam sheets were provided in the whirligig tank for female whirligig beetles to lay eggs underneath them. After hatching, whirligig larvae swim to the bottom of the aquarium where they remain for most of their larval life. The total developmental time before adult emergence is about 6 weeks, including a week each in the egg and the 1^st^, 2^nd^ and 3^rd^ instar larval stages, in addition to 2 weeks of pupation. Right before pupation, the last (3^rd^) instar larva swims to the water surface and searches for a suitable terrestrial substrate on the riverbank. There it builds a sandy chamber (puparium) and pupates. In the present study, wandering larvae were transferred to another tank with shallow water and sand. Each larva uses the sand to build a puparium where it encloses itself and then pupates. Adult beetles, 3^rd^ instar larvae and selected ages of whirligig pupae were used for the present account.

### Bodian reduced silver staining

Eight whirligig adults and 10 3^rd^ instar larvae were used for silver staining following the procedures described previously [11]. In brief, the insects were cold anesthetized, the heads opened, fixed in AAF (17 ml 100% ethanol, 1 ml glacial acetic acid, 2 ml 37% formaldehyde), and the brains then freed from the head capsule. Brains were embedded in Paraplast Plus (Tyco, Mansfield, Mass.) and serially sectioned at 8–12 μm before processing using Bodian’s original method [31].

### Immunohistochemistry

Five adult whirligig and 5 adult sunburst diving beetles were used for comparative immunolabeling of their optic lobes, following the procedures described previously [32]. In brief, brains were fixed overnight in 4% paraformaldehyde in phosphate buffer (pH 7.4), and then washed in phosphate-buffered saline (PBS), embedded in albumin gelatin, and sectioned at 60 μm with a vibratome (Leica, Nussloch, Germany). After being washed with PBS-TX (0.5% Triton X-100 in PBS), sections were blocked in 5% normal donkey serum (Jackson Immunoresearch Laboratories, West Grove, Penn.) for 1 hr, and then incubated overnight in monoclonal α-tubulin antiserum (1: 100; Developmental Studies Hybridoma Bank, University of Iowa, Iowa) on a shaker at room temperature. The following day, sections were washed with PBS-TX and incubated overnight in the secondary donkey anti-mouse immunoglobulins conjugated to Cy3 (dilution 3: 1000; Jackson Immunoresearch Laboratories, West Grove, Penn.). The following day, sections were washed with PBS, incubated in the fluorescent nuclear stain Syto-13 (dilution 1: 4,000; Life Technologies, Grand Island, N.Y.) for 20 min, washed with Tris–HCl buffer (pH 7.4), and mounted on slides and coverslipped in a medium of 25% polyvinyl alcohol, 25% glycerol and 50% PBS.

### Imaging and reconstructions

Images of Bodian preparations were digitally collected using a Zeiss Axio Imager Z2 microscope with AxioVision software. Broad area images were reconstructed at 20x, using optical sections taken in 0.5-1 μm steps through the section. Each layer was stitched with the adjacent fields and then montaged images were automatically reconstructed in the z-plane using Helicon Focus (Helicon Soft, Kharkov, Ukraine). Confocal reconstructions of immunolabeled optic lobes were made with an LSM 5 Pascal confocal microscope (Zeiss, Oberkochen, Germany). Images of 1,024 x 1,024 pixel resolution at 12-bit color depth were scanned using a 20/0.5 plan Neofluar objective. Selected images were digitally assembled and adjusted for brightness and contrast using Adobe Photoshop CS3 (Adobe Systems, San Jose, Calif.). Pictures of whirligig larvae were collected with a digital camera (Sony DSC-W80) connected to a dissecting microscope. Schematic illustrations were made using Adobe Illustrator CS3 (Adobe Systems, San Jose, Calif.).

## Competing interests

The authors declare that they have no competing interests.

## Authors’ contributions

NJS and CL designed the experiments. CL carried out the study. CL and NJS wrote the manuscript. Both authors read and approved the final manuscript.
